# Development and temporal validation of a nomogram for estimating the risk of malignancy in surgically managed adnexal masses after hysterectomy

**DOI:** 10.3389/fmed.2026.1915489

**Published:** 2026-10-05

**Authors:** Le Xin, Anrui Yang, Ming Wang, Ying Zhang

**Affiliations:** 1 Department of Gynecological Minimal Invasive Center, Beijing Obstetrics and Gynecology Hospital, Capital Medical University, Beijing Maternal and Child Health Care Hospital, Beijing, China; 2 Department of Gynecologic Oncology, Beijing Obstetrics and Gynecology Hospital, Laboratory for Clinical Medicine, Capital Medical University, Beijing Maternal and Child Health Care Hospital, Beijing, China

**Keywords:** adnexal mass, hysterectomy, malignancy, nomogram, risk prediction, temporal validation, ultrasonography

## Abstract

**Background:**

Adnexal masses discovered after hysterectomy are common, but the proportion that is malignant is low, and existing risk-stratification systems such as O-RADS were not developed or validated in this population. We aimed to develop and temporally validate a nomogram estimating the probability of malignancy among patients who have already been selected for surgical management of a post-hysterectomy adnexal mass.

**Methods:**

We retrospectively studied consecutive patients who underwent re-operation for an adnexal mass after prior benign hysterectomy at Beijing Obstetrics and Gynecology Hospital between January 2010 and October 2025. Patients operated from January 2010 to September 2023 formed the development cohort (*n* = 233; 50 malignant, 183 benign); those operated from October 2023 to October 2025 formed the temporal validation cohort (*n* = 48; 20 malignant, 28 benign). Fourteen candidate predictors were screened by LASSO regression with 10-fold cross-validation. Final coefficients were obtained from unpenalised logistic regression on the selected predictors; the intercept of the seven-variable model was recalibrated on the development cohort because the originally reported intercept was inconsistent with the scaling of the continuous predictors. Performance was assessed by discrimination, calibration (intercept, slope, Brier score) and decision-curve analysis, with 1,000 bootstrap resamples for optimism correction.

**Results:**

The seven-variable nomogram comprises age (per 10 years), BMI (per 10 kg/m^2^), tumour size (ordinal), cystic components, enriched blood flow/enhancement, elevated CA125 (≥70 U/mL) and cyst duration (per 5 years). In the development cohort the AUC was 0.959 (95% CI 0.922–0.990) for the seven-variable model and 0.937 (0.885–0.979) for a simplified three-variable model (cystic components, enhancement, elevated CA125); the difference favoured the full model (*Δ*AUC −0.023, 95% CI −0.045 to −0.005; DeLong *p* = 0.021). In the temporal validation cohort the AUC was 0.905 (0.791–0.985) and 0.929 (0.846–0.986), respectively (*Δ*AUC +0.022, 95% CI −0.040 to 0.097; *p* = 0.517). Calibration was adequate in development (intercept 0.00, slope 1.02) and showed no statistically significant miscalibration in validation (intercept 0.29, 95% CI −0.71 to 1.28; slope 0.71, 95% CI 0.34 to 1.08). At the pre-specified 0.5 threshold the full model missed 8 of 50 malignancies in development and 6 of 20 in validation.

**Conclusion:**

We present a parsimonious nomogram that estimates malignancy risk in post-hysterectomy adnexal masses among patients already selected for surgery, with internal and temporal validation. Because all participants underwent surgery, the model cannot be used to justify non-operative management; independent external and prospective validation in unselected cohorts is required before any clinical implementation.

## 1 Introduction

Adnexal masses detected after hysterectomy pose a distinct diagnostic problem. Hysterectomy is performed in approximately 1.2 million women annually in the United States alone and substantially alters pelvic anatomy; the reported incidence of pelvic masses after hysterectomy reaches 50.7%, with re-operation rates of 2.7%–5.5% (1, 2). Only about 18% of patients who undergo re-operation are found to have a malignant mass (3). Postoperative adhesions, altered vascular supply and the absence of uterine landmarks all reduce the reliability of sonographic assessment, and clinicians frequently proceed to surgery because pre-operative risk cannot be quantified.

Established prediction tools, including the Ovarian-Adnexal Reporting and Data System (O-RADS) (4, 5), the IOTA logistic regression models and the ADNEX model (6, 7), were developed and validated in populations with an intact uterus. Women with prior hysterectomy were not represented in those cohorts, so their applicability here is extrapolative rather than demonstrated (5, 8, 9). Interpretation of tumour markers may also differ: benign uterine sources of CA125 elevation such as adenomyosis and leiomyomas are removed at hysterectomy, which could alter the specificity of CA125 in this setting.

We therefore aimed (i) to identify predictors of malignancy specific to the post-hysterectomy setting, (ii) to develop and temporally validate a dedicated nomogram, and (iii) to derive a simplified version suitable for bedside use. We emphasise that our cohorts consist exclusively of patients who underwent surgery; the model is intended for pre-operative risk estimation in surgical candidates and not for deciding whether surgery can be safely avoided.

## 2 Materials and methods

### 2.1 Study population and design

This retrospective cohort study included consecutive patients who underwent surgical evaluation for an adnexal mass following prior hysterectomy at Beijing Obstetrics and Gynecology Hospital between January 2010 and October 2025. The study was approved by the Ethics Committee of Beijing Obstetrics and Gynecology Hospital, Capital Medical University (approval 2024-KY-049-01) and conducted in accordance with the Declaration of Helsinki; informed consent was waived because of the retrospective design and the use of de-identified data.

Inclusion criteria were: documented hysterectomy for benign disease; a newly diagnosed adnexal mass requiring surgical intervention; complete pre-operative imaging (transvaginal ultrasonography and/or CT/MRI); and serum CA125 measurement within 30 days before surgery. Exclusion criteria were: previous ovarian malignancy or borderline tumour; hysterectomy performed for malignant disease; and an imaging-to-surgery interval exceeding 120 days. The unit of analysis was the patient. When more than one adnexal lesion was present, imaging predictors were taken from the lesion with the most suspicious ultrasonographic morphology.

Patients operated between January 2010 and September 2023 constituted the development cohort; patients operated between October 2023 and October 2025 constituted the temporal validation cohort. The split was defined by calendar period of surgery, and the validation data were not used for any step of predictor selection or model fitting. The validation cohort was ascertained retrospectively from existing electronic medical records after the analysis plan had been fixed; no prospective enrolment was performed. Throughout this paper we therefore use the term “temporal validation” rather than “external validation”. The study is reported in accordance with TRIPOD (10).

### 2.2 Predictor definitions and imaging assessment

Fourteen candidate predictors were considered (Supplementary Table S3). Definitions relevant to replication are given here.

Elevated tumour marker was defined as pre-operative serum CA125 ≥ 70 U/mL (twice the upper limit of normal at our institution), measured with a chemiluminescent immunoassay on the same platform family throughout the study period, with a single prespecified threshold applied to all patients irrespective of menopausal status. CA125 and HE4 were not combined into a composite variable: HE4 was measured in only a subset of patients and was not retained in the final model. CA19-9 and CEA were available only when selectively requested and were therefore excluded from the candidate set.

Enhancement/enriched blood flow was scored on colour-Doppler ultrasonography as an ordinal variable: 0 = no detectable flow or no contrast enhancement; 1 = dotted or mild flow/mild enhancement; 2 = rich, clearly enriched flow or strong enhancement. Ultrasonography was performed in all patients and was the primary source for this variable; CT or MRI contrast enhancement was used only as a substitute when Doppler assessment was technically inadequate, and was mapped onto the same three categories.

Cystic components were coded 0 = purely cystic; 1 = cystic-solid, including lesions with mural papillary projections; 2 = solid. Cyst duration was defined as the interval in years between the first radiological detection of the mass after hysterectomy and the index re-operation, obtained from historical imaging records.

Two radiologists with subspecialty experience in gynaecological imaging independently reviewed all images, blinded to pathological results, tumour-marker values and clinical information; disagreements were resolved by consensus. Inter-observer agreement was not quantified in this study and will be assessed in future external cohorts.

### 2.3 Outcome

The outcome was the histological diagnosis of the surgical specimen. Malignant lesions comprised invasive epithelial ovarian carcinomas (high-grade serous, low-grade serous, endometrioid and clear cell), fallopian-tube and primary peritoneal carcinomas, malignant tumours metastatic to the adnexa, and borderline ovarian tumours. Borderline tumours were analysed within the malignant category because they generally require oncological surgical management; their number is reported separately in Supplementary Table S4. All specimens were examined by pathologists at our institution according to the WHO classification current at the time of diagnosis.

### 2.4 Statistical methods

Continuous variables are summarised as median (interquartile range) and compared with the Mann–Whitney U test; categorical variables are summarised as counts (percentages) and compared with the chi-square test without continuity correction, or with Fisher’s exact test (2 × 2 tables) or a Monte-Carlo permutation test (20,000 replicates, r × c tables) when expected cell counts were below five.

Candidate predictors were screened by LASSO logistic regression with 10-fold cross-validation (glmnet), using lambda.min to define the full model and lambda.1se to define the simplified model. Predictors were not standardised, a fixed random seed was set, and fold allocation followed the glmnet default. Final coefficients were obtained from unpenalised multivariable logistic regression fitted on the LASSO-selected predictors; LASSO was used for screening only and did not shrink the coefficients of the reported model.

Linearity of continuous predictors was examined with restricted cubic splines (four knots at the 5th, 35th, 65th and 95th centiles), adjusted for the remaining predictors of the full model, using Wald tests of the non-linear terms (Supplementary Figure S2). For the three-level ordinal size variable, the ordinal (linear-in-category) coding was compared with unconstrained dummy coding by a likelihood-ratio test.

Internal validation used 1,000 bootstrap resamples for optimism correction, performed after variable selection on the fixed predictor set; we therefore report the resulting shrinkage factor descriptively and regard temporal validation as the primary evidence against substantial overfitting. Discrimination is reported as the area under the receiver operating characteristic curve (AUC) with bootstrap confidence intervals; model comparison used the DeLong test with bootstrap confidence intervals for the AUC difference. Calibration is reported as the calibration intercept (calibration-in-the-large), calibration slope and Brier score with 95% confidence intervals, and is displayed as separate calibration plots for each cohort. Decision-curve analysis was performed separately in each cohort over threshold probabilities of 0.01–0.89; net benefit was not standardised and confidence intervals were not computed.

Missing data were minimal. In the development cohort, age was missing for one patient, BMI for four patients and cyst duration for two patients; in the validation cohort tumour size was missing for one patient. These values were replaced by the cohort median, and a complete-case sensitivity analysis was performed. Analyses were performed in R (glmnet, rms, pROC, rmda) and independently reproduced in Python (statsmodels); both implementations produced identical estimates.

## 3 Results

### 3.1 Study population

Of 308 patients assessed for eligibility, 27 were excluded because of incomplete pre-operative imaging or the absence of a tumour-marker result within 30 days, leaving 281 patients (Supplementary Figure S1): 233 in the development cohort (50 malignant, 21.5%) and 48 in the temporal validation cohort (20 malignant, 41.7%). Baseline characteristics of both cohorts, stratified by final histological diagnosis, are shown in Table 1, and a direct comparison of the two periods is given in Supplementary Table S2.

**Table 1:** Baseline characteristics of the study population stratified by cohort and histological diagnosis.

Variable	Overall Benign (*n* = 211)	Overall Malignant (*n* = 69)	Overall P	Development Benign (*n* = 183)	Development Malignant (*n* = 50)	Development P	Validation Benign (*n* = 28)	Validation Malignant (*n* = 20)	Validation P
Age (years)	53.0 [48.0; 60.0]	59.0 [53.0; 63.0]	0.001	53.0 [48.0; 58.0]	57.0 [52.2; 61.0]	0.011	58.0 [54.0; 65.0]	62.5 [58.0; 66.0]	0.330
Age group			0.031			0.169			0.535
< 50 years	63 (29.9%)	12 (17.1%)		60 (32.8%)	10 (20.0%)		3 (10.7%)	2 (10.0%)	
50–<60 years	94 (44.5%)	30 (42.9%)		81 (44.3%)	24 (48.0%)		13 (46.4%)	6 (30.0%)	
≥ 60 years	54 (25.6%)	28 (40.0%)		42 (23.0%)	16 (32.0%)		12 (42.9%)	12 (60.0%)	
Gravidity (n)	2 [2; 3]	2 [1; 3]	0.364	2 [2; 3]	2 [1; 3]	0.146	2 [2; 3]	2 [2; 3]	0.504
Parity (n)	1 [1; 1]	1 [1; 1]	0.730	1 [1; 1]	1 [1; 1]	0.605	1 [1; 1]	1 [1; 1]	0.792
Family history of cancer			0.235			0.126			0.915
Unknown	7 (3.3%)	1 (1.4%)		7 (3.8%)	1 (2.0%)		0 (0.0%)	0 (0.0%)	
None	133 (63.0%)	38 (54.3%)		113 (61.7%)	24 (48.0%)		20 (71.4%)	14 (70.0%)	
Positive	71 (33.6%)	31 (44.3%)		63 (34.4%)	25 (50.0%)		8 (28.6%)	6 (30.0%)	
BMI (kg/m^2^)	25.4 [23.0; 27.9]	25.3 [22.7; 27.5]	0.932	25.3 [23.0; 27.7]	25.4 [23.3; 27.7]	0.551	26.1 [23.5; 29.7]	24.4 [22.4; 27.3]	0.188
BMI ≥28 kg/m^2^			0.427			0.887			0.111
Yes	52 (24.6%)	14 (20.0%)		42 (23.0%)	11 (22.0%)		10 (35.7%)	3 (15.0%)	
No	159 (75.4%)	56 (80.0%)		141 (77.0%)	39 (78.0%)		18 (64.3%)	17 (85.0%)	
Tumour size			<0.001			<0.001			0.931
< 5 cm	21 (10.0%)	16 (22.9%)		15 (8.2%)	11 (22.0%)		6 (21.4%)	5 (25.0%)	
5–<10 cm	126 (59.7%)	19 (27.1%)		116 (63.4%)	13 (26.0%)		10 (35.7%)	6 (30.0%)	
≥ 10 cm	64 (30.3%)	35 (50.0%)		52 (28.4%)	26 (52.0%)		12 (42.9%)	9 (45.0%)	
Cystic components			<0.001			<0.001			<0.001
Cystic	170 (80.6%)	9 (12.9%)		146 (79.8%)	6 (12.0%)		24 (85.7%)	3 (15.0%)	
Cystic-solid/mural papilla	10 (4.7%)	21 (30.0%)		8 (4.4%)	7 (14.0%)		2 (7.1%)	14 (70.0%)	
Solid	31 (14.7%)	40 (57.1%)		29 (15.8%)	37 (74.0%)		2 (7.1%)	3 (15.0%)	
Multilocular cyst			<0.001			<0.001			0.031
Yes	122 (57.8%)	14 (20.0%)		111 (60.7%)	13 (26.0%)		11 (39.3%)	1 (5.0%)	
No	88 (41.7%)	51 (72.9%)		72 (39.3%)	37 (74.0%)		16 (57.1%)	14 (70.0%)	
Missing	1 (0.5%)	5 (7.1%)		0 (0.0%)	0 (0.0%)		1 (3.6%)	5 (25.0%)	
Enhancement/blood flow			<0.001			<0.001			<0.001
None	174 (82.5%)	11 (15.7%)		147 (80.3%)	7 (14.0%)		27 (96.4%)	4 (20.0%)	
Dotted/mild	17 (8.1%)	4 (5.7%)		16 (8.7%)	3 (6.0%)		1 (3.6%)	1 (5.0%)	
Enriched/strong	20 (9.5%)	55 (78.6%)		20 (10.9%)	40 (80.0%)		0 (0.0%)	15 (75.0%)	
CA125 ≥ 70 U/mL			<0.001			<0.001			<0.001
Yes	11 (5.2%)	53 (75.7%)		6 (3.3%)	39 (78.0%)		5 (17.9%)	14 (70.0%)	
No	200 (94.8%)	17 (24.3%)		177 (96.7%)	11 (22.0%)		23 (82.1%)	6 (30.0%)	
Cyst duration (years)	9.0 [2.0; 14.5]	11.0 [6.0; 17.0]	0.038	7.0 [2.0; 14.0]	9.5 [5.0; 13.0]	0.143	14.0 [10.0; 17.2]	17.5 [7.5; 20.5]	0.432
Cyst duration group			0.014			0.047			0.345
< 5 years	74 (35.1%)	12 (17.1%)		74 (40.4%)	11 (22.0%)		0 (0.0%)	1 (5.0%)	
5–<10 years	38 (18.0%)	19 (27.1%)		34 (18.6%)	14 (28.0%)		4 (14.3%)	5 (25.0%)	
≥ 10 years	99 (46.9%)	39 (55.7%)		75 (41.0%)	25 (50.0%)		24 (85.7%)	14 (70.0%)	
Fallopian tubes preserved			0.362			0.380			0.683
Yes	184 (87.2%)	58 (82.9%)		159 (86.9%)	41 (82.0%)		25 (89.3%)	17 (85.0%)	
No	27 (12.8%)	12 (17.1%)		24 (13.1%)	9 (18.0%)		3 (10.7%)	3 (15.0%)	

Data are presented as median [IQR] for continuous variables and *n* (%) for categorical variables; percentages are calculated within the benign or malignant column of each cohort. *P* values compare benign with malignant lesions within each cohort and were obtained from the Mann–Whitney U test for continuous variables and the chi-square test (without continuity correction), Fisher's exact test (2 × 2 tables with expected counts < 5) or a Monte-Carlo permutation test (20,000 replicates; r × c tables with expected counts < 5) for categorical variables. The temporal validation cohort comprises 48 patients (28 benign, 20 malignant); all descriptive statistics in Table 1 and all model-performance analyses use the full 48 patients. History of endometriosis and accompanying metastatic disease on imaging were not retrievable for the temporal validation cohort and are therefore not shown; neither variable entered either final model. Multilocular status was coded 2 (“no ultrasound description”) for six validation patients and is treated as missing for those patients.

In both cohorts, malignant lesions occurred in older patients (overall median 59 vs. 53 years, *p* < 0.001), were more often solid or cystic-solid with enriched blood flow, less often multilocular, and were far more frequently associated with CA125 ≥ 70 U/mL (overall 76.8% vs. 4.8%, *p* < 0.001). Compared with the development cohort, patients in the validation period were older (median 60 vs. 53 years, *p* < 0.001), had a longer interval from hysterectomy to detection of the mass (median 15 vs. 8 years, *p* < 0.001), more often had cystic-solid morphology (33.3% vs. 6.4%) and elevated CA125 (39.6% vs. 19.3%, *p* = 0.002), and had a higher prevalence of malignancy (41.7% vs. 21.5%, *p* = 0.003). These differences are relevant to the interpretation of the validation results and are discussed below.

### 3.2 Predictor selection and model specification

LASSO regression with 10-fold cross-validation retained seven of the fourteen candidate predictors at lambda.min: age, BMI, tumour size, cystic components, enhancement/enriched blood flow, elevated CA125 and cyst duration. At lambda.1se three predictors remained: cystic components, enhancement/enriched blood flow and elevated CA125 (Figure 1). Coefficients of the two models, obtained from unpenalised logistic regression, are shown in Table 2. Retention by LASSO does not imply statistical significance; in the unpenalised model only enhancement (*p* = 0.001) and CA125 (*p* < 0.001) reached conventional significance.

**Figure 1:**
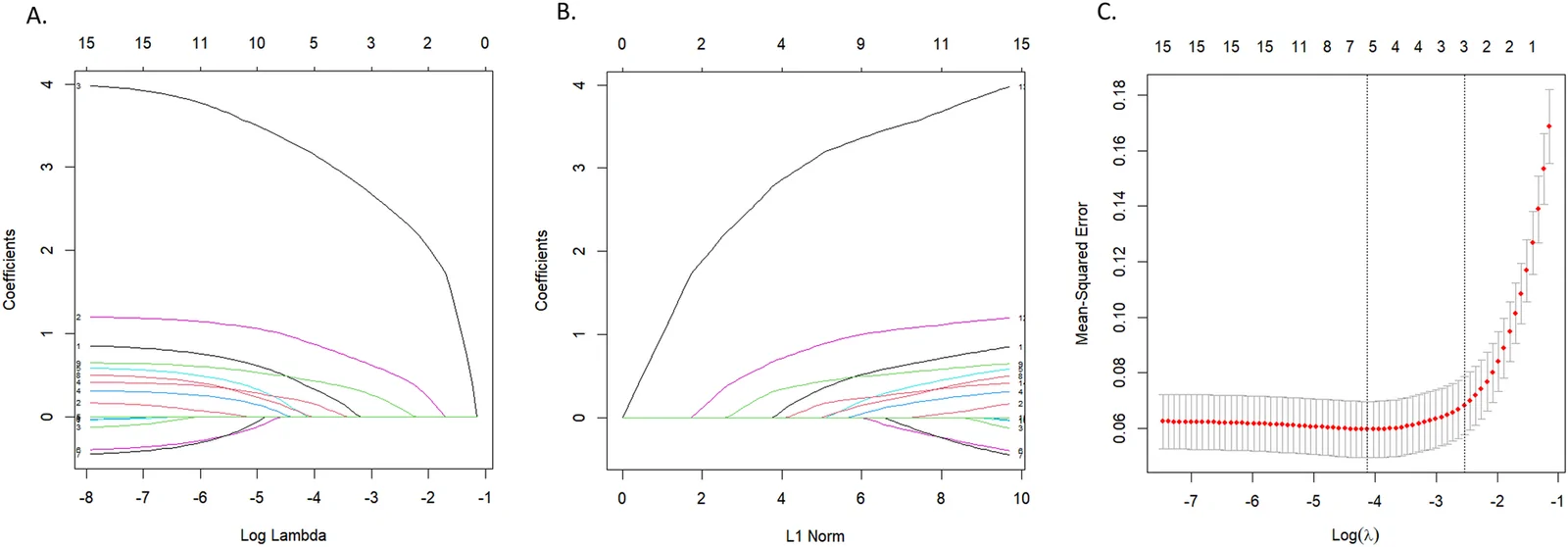
Predictor selection by LASSO logistic regression with 10-fold cross-validation (development cohort, *n* = 233). **(A)** Coefficient paths for all 14 candidate predictors plotted against log(*λ*); each path is labelled with the corresponding predictor and the number of predictors retained at each value is indicated along the top. **(B)** Coefficient paths plotted against the L1 norm. **(C)** Cross-validated mean squared error (mean ± 1 SD) plotted against log(*λ*); the vertical dotted lines mark lambda.min (*λ* = 0.00036, log *λ* = −3.44), the value giving minimum cross-validated error, and lambda.1se, the largest *λ* within one standard error of the minimum. Seven predictors were retained at lambda.min and three at lambda.1se.

**Table 2:** Coefficients of the final models (development cohort, *n* = 233).

Predictor	Coefficient (*β*)	Odds ratio	95% CI	*P* value
Intercept	−12.990	—	—	<0.001
Age (per 10 years)	0.934	2.55	0.95–6.83	0.064
BMI (per 10 kg/m^2^)	0.699	2.01	0.50–8.04	0.322
Tumour size (ordinal, per category)	0.401	1.49	0.60–3.75	0.392
Cystic components (0/1/2)	0.568	1.76	0.86–3.61	0.120
Enhancement/blood flow (0/1/2)	1.292	3.64	1.69–7.86	0.001
CA125 ≥ 70 U/mL	3.970	53.01	12.85–218.7	<0.001
Cyst duration (per 5 years)	0.373	1.45	0.67–3.17	0.349
Panel B: simplified 3-variable model				
Intercept	−3.959	—	—	<0.001
Cystic components (0/1/2)	0.636	1.89	0.96–3.71	0.065
Enhancement/blood flow (0/1/2)	1.002	2.72	1.38–5.38	0.004
CA125 ≥ 70 U/mL	3.582	35.94	10.97–117.7	<0.001

The intercept of the seven-variable model was recalibrated on the development cohort (−12.990) because the intercept originally reported (−6.757) was incompatible with the scaling of the continuous predictors used in the published equation; recalibration does not change discrimination. The full equation, predictor coding and a worked example are given in Supplementary Table S1.

### 3.3 Nomogram

Figure 2 shows the full seven-variable nomogram and the simplified three-variable nomogram. In both, elevated CA125 carries the largest weight, followed by enhancement/enriched blood flow and cystic components.

**Figure 2:**
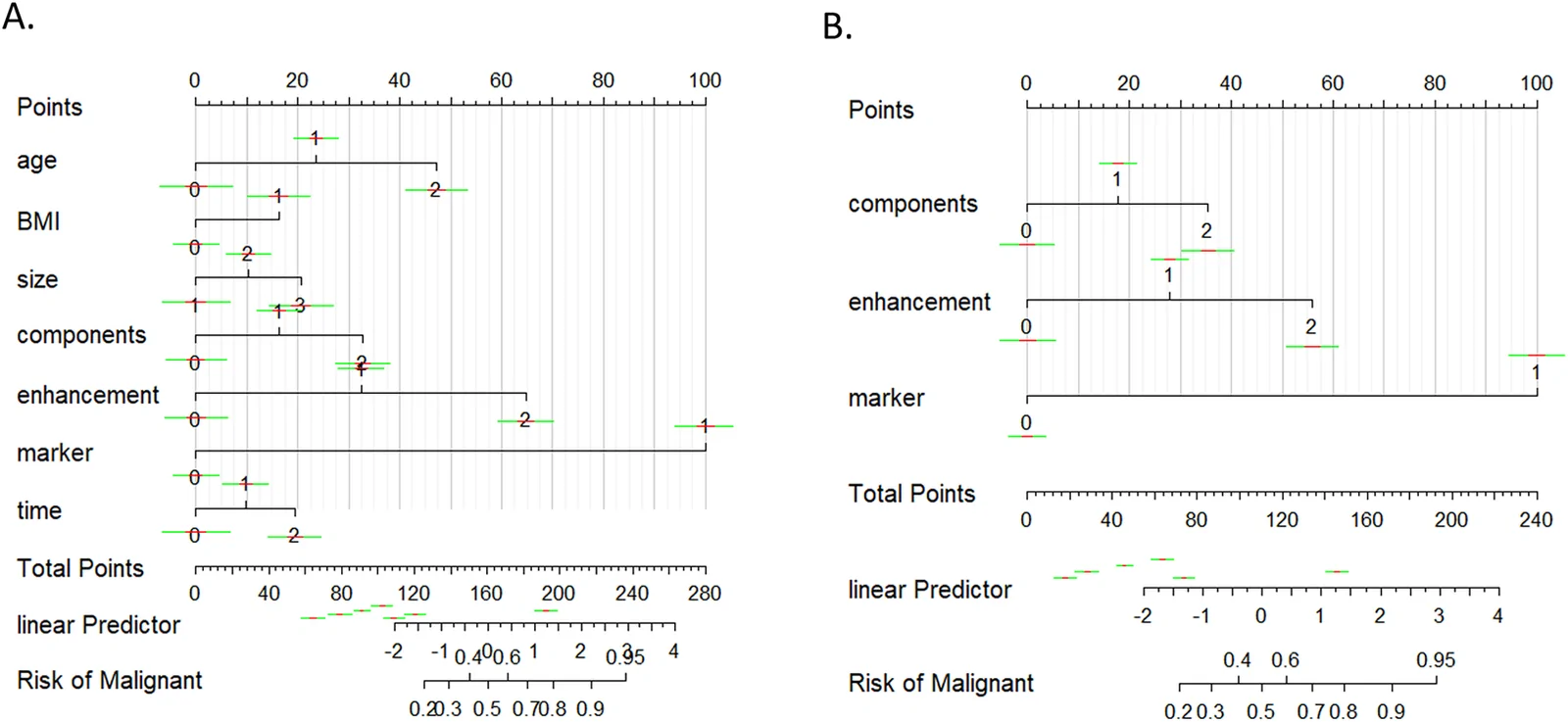
Nomograms for estimating the probability of malignancy in post-hysterectomy adnexal masses. **(A)** Full seven-variable nomogram: age (years, continuous axis), BMI (kg/m^2^, continuous axis), tumour size (0 = <5 cm, 1 = 5–<10 cm, 2 = ≥10 cm), cystic components (0 = purely cystic, 1 = cystic-solid or mural papillary projections, 2 = solid), enhancement/blood flow (0 = none, 1 = dotted or mild, 2 = enriched or strong), CA125 (0 = <70 U/mL, 1 = ≥70 U/mL) and cyst duration (years). **(B)** Simplified three-variable nomogram comprising cystic components, enhancement/blood flow and CA125, with the same category definitions and units. To use either nomogram, locate each predictor on its axis, read the corresponding points, sum them and read the total-points axis to obtain the predicted probability of malignancy.

### 3.4 Discrimination, calibration and clinical utility

Discrimination, calibration and threshold-based performance are summarised in Supplementary Table S5 and the ROC curves are shown in Figure 3. In the development cohort, AUC was 0.959 (95% CI 0.922–0.990) for the seven-variable model and 0.937 (0.885–0.979) for the three-variable model; the difference favoured the full model (*Δ*AUC −0.023, 95% CI −0.045 to −0.005; DeLong Z = −2.32, *p* = 0.021). In the temporal validation cohort, AUC was 0.905 (0.791–0.985) and 0.929 (0.846–0.986), respectively (*Δ*AUC +0.022, 95% CI −0.040 to 0.097; Z = 0.65, *p* = 0.517). The confidence intervals for the validation difference are wide and include both a modest advantage and a modest disadvantage of the simplified model; we therefore do not claim equivalence.

**Figure 3:**
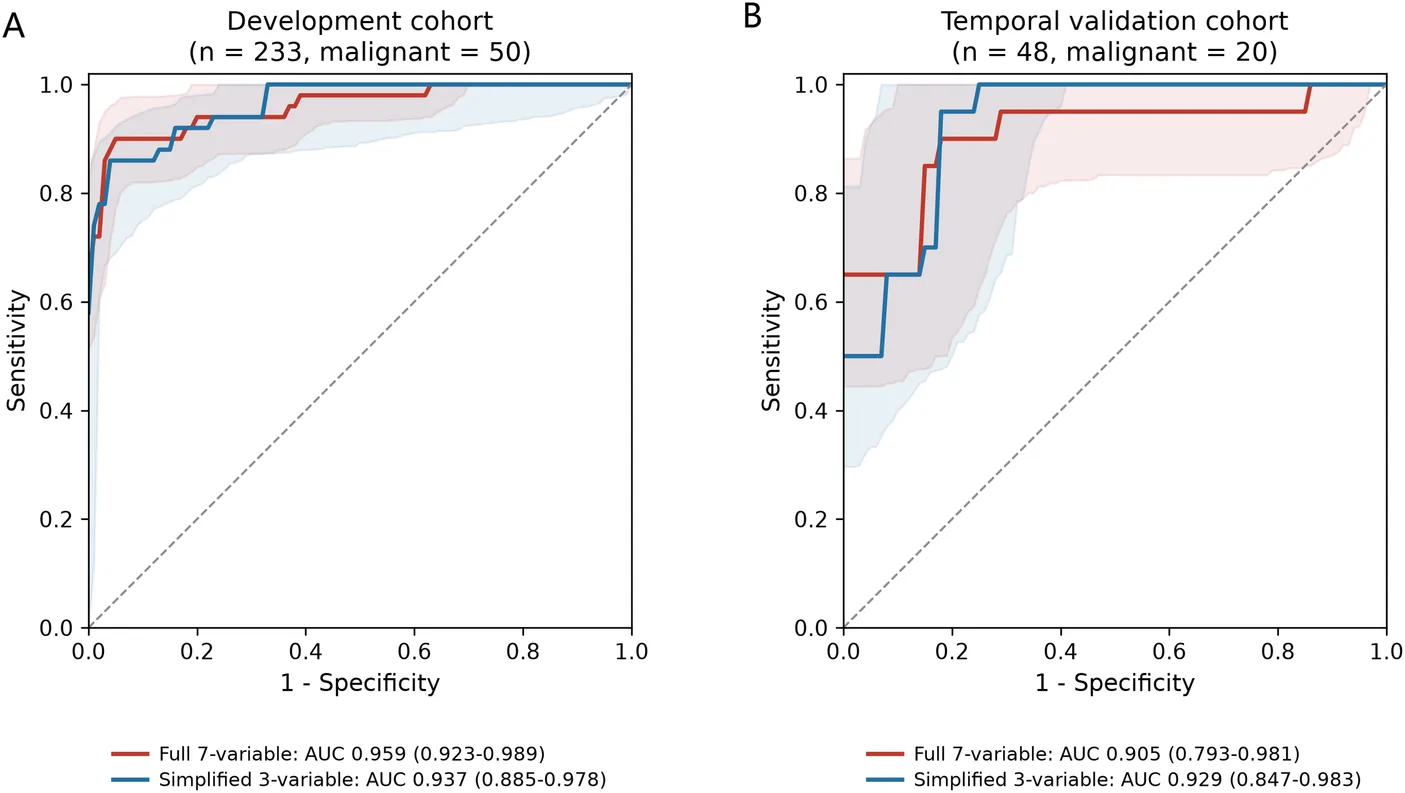
Receiver operating characteristic curves of the seven-variable nomogram and the simplified three-variable model. **(A)** Development cohort (*n* = 233; 50 malignant). **(B)** Temporal validation cohort (*n* = 48; 20 malignant). Shaded areas are 95% bootstrap confidence bands; AUC values with 95% confidence intervals are given in each panel. The dashed diagonal represents chance performance.

Calibration of the seven-variable model was good in the development cohort (intercept 0.00, 95% CI −0.57 to 0.57; slope 1.02, 95% CI 0.74 to 1.30; Brier 0.047) and acceptable in the temporal validation cohort (intercept 0.29, 95% CI −0.71 to 1.28, *p* = 0.57; slope 0.71, 95% CI 0.34 to 1.08; Brier 0.121), where the slope below unity with a wide interval indicates that some over-fitting of the predictor weights cannot be excluded (Supplementary Figures S5 and S6).

At the pre-specified threshold of 0.5, the seven-variable model detected 42 of 50 malignancies (sensitivity 84.0%, 95% CI 71.5–91.7%) with specificity 97.3% (93.8–98.8%) in the development cohort, and 14 of 20 malignancies (sensitivity 70.0%, 95% CI 48.1–85.5%) with specificity 85.7% (68.5–94.3%) in the validation cohort. Results at the exploratory 0.1 threshold and at Youden-optimal thresholds, both of which are reported for completeness and not as recommended decision boundaries, are given in Supplementary Table S5. Decision-curve analysis showed a net benefit above both treat-none and treat-all across threshold probabilities of approximately 0.1 to 0.7 in the development cohort and 0.1 to 0.6 in the validation cohort (Supplementary Figures S3 and S4).

Bootstrap optimism correction (1,000 resamples) gave corrected C-indices of 0.93 for the full model and 0.95 for the simplified model with a shrinkage factor of 0.993. Because resampling was performed on a fixed predictor set rather than repeating the entire selection procedure, this value is reported descriptively.

### 3.5 Linearity, benchmark comparisons and sensitivity analyses

Restricted cubic spline analysis supported linearity for BMI (*p* = 0.758) and cyst duration (*p* = 0.651) but not for age (*p* = 0.002), whose fitted curve was unstable at the extremes of the age distribution with very wide confidence intervals (Supplementary Figure S2). For tumour size, the ordinal coding fitted significantly worse than unconstrained dummy coding (likelihood-ratio *χ*^2^ = 7.34, df = 1, *p* = 0.007): observed malignant proportions were 42.3% (11/26) for masses <5 cm, 10.1% (13/129) for 5–<10 cm and 33.3% (26/78) for ≥10 cm, a U-shaped pattern that a single linear term cannot represent. Small masses are likely to be solid lesions detected because of symptoms rather than size.

We nevertheless retained the pre-specified parsimonious coding. A flexible model allowing a spline for age and unconstrained categories for size increased apparent discrimination in the development cohort (AUC 0.982 vs. 0.959) but decreased discrimination in the temporal validation cohort (AUC 0.880 vs. 0.905), the pattern expected when additional flexibility captures noise rather than signal.

To place the incremental value of the nomogram in context, we fitted four benchmark models (Table 3). A model using elevated CA125 alone already achieved an AUC of 0.874 (0.814–0.931) in the development cohort and 0.761 (0.628–0.880) in the validation cohort, confirming that most of the discriminative information resides in this single variable; the full nomogram adds a modest increment. A clinical model without biomarker information performed poorly (AUC 0.624 and 0.511).

**Table 3:** Benchmark comparisons (AUC with 95% CI).

Model	Development cohort (*n* = 233)	Temporal validation cohort (*n* = 48)
Elevated CA125 alone (≥70 U/mL)	0.874 (0.814–0.931)	0.761 (0.628–0.880)
Imaging morphology alone (cystic components)	0.844 (0.786–0.895)	0.834 (0.708–0.941)
Enriched vascularity alone	0.863 (0.801–0.917)	0.896 (0.799–0.976)
Clinical model without biomarker (age, BMI, size, duration)	0.624 (0.532–0.714)	0.511 (0.334–0.686)
Simplified 3-variable model	0.937 (0.885–0.979)	0.929 (0.846–0.986)
Full 7-variable nomogram	0.959 (0.922–0.990)	0.905 (0.791–0.985)

A complete-case sensitivity analysis that excluded every patient with an imputed value (development *n* = 227 with 47 events; validation *n* = 47 with 19 events) gave AUCs of 0.965 (seven-variable) and 0.946 (three-variable) in development and 0.900 and 0.925 in validation, closely similar to the primary analysis.

### 3.6 Histological diagnoses

Complete histological diagnoses for both cohorts are listed in Supplementary Table S4. In the development cohort the 50 malignant lesions comprised 26 high-grade serous carcinomas, 10 endometriosis-associated carcinomas, 6 borderline tumours and 8 other malignancies; the 183 benign lesions comprised 120 benign teratomas, 54 chronic pelvic inflammatory lesions and 9 myomas or other benign lesions. In the validation cohort the 20 malignant lesions comprised 8 high-grade serous carcinomas, 2 low-grade serous carcinomas, 2 endometrioid carcinomas, 1 clear-cell carcinoma, 4 borderline tumours, 2 tubal adenocarcinomas and 1 adenocarcinoma of the vaginal vault; the 28 benign lesions were predominantly serous (*n* = 7), mucinous (*n* = 5), seromucinous (*n* = 3) and endometriotic cysts (*n* = 4).

## 4 Discussion

We developed and temporally validated a nomogram that estimates the probability of malignancy in adnexal masses detected after hysterectomy among patients who have already been selected for surgery. Two findings deserve emphasis. First, a three-predictor model based on morphology, vascularity and CA125 achieves discrimination close to that of the seven-predictor model and may be preferable in busy practice. Second, and more cautiously, most of the discriminative information in this cohort is carried by CA125 alone; the incremental contribution of the remaining predictors is real but modest.

The suggestion that hysterectomy provides a “cleansed diagnostic background” that improves the specificity of CA125 — because common benign uterine sources of CA125 elevation have been removed — remains a hypothesis. Our data contain no comparison with women who have not undergone hysterectomy, and we cannot establish that the observed performance of CA125 is attributable to the elimination of adenomyosis or leiomyomas. We present this interpretation as a possible biological explanation requiring direct comparative study rather than as a demonstrated conclusion.

Solid components and enriched blood flow are established markers of malignancy in adnexal lesions and retained their importance here (11–14). Our observation that very small masses carry a relatively high risk of malignancy is consistent with the view that small solid lesions often present because of symptoms or suspicious morphology rather than through size-related detection, and warrants confirmation in larger cohorts.

Relative to categorical ultrasound-based systems such as O-RADS (4, 5, 15) and the ADNEX model (7), our instrument adds simple clinical variables and returns a continuous individualised probability rather than a risk category, and it was developed in the population to which it applies. These are potential rather than demonstrated advantages: we did not perform a head-to-head comparison with O-RADS or ADNEX, because the required prospective scoring was not recorded, and we make no claim of superiority over existing tools.

Strengths of this study include the use of histopathology as the reference standard, the use of a temporal rather than random split, the assessment of discrimination, calibration and decision-analytic performance rather than AUC alone, and transparent reporting of a locked, reproducible model.

Limitations must be stated plainly. First, all participants underwent surgery, so selection, referral and spectrum bias are unavoidable; the model cannot support the conclusion that patients below any given predicted risk can safely avoid surgery, and statements about avoiding surgery are hypotheses requiring prospective evaluation in unselected cohorts that include conservatively managed patients. Second, this was a single-centre study, and independent external validation in centres with different prevalence, imaging expertise and laboratory methods is required before clinical use. Third, the seven-variable model has an events-per-parameter ratio of 7.14 (50 events, 7 parameters), below the conventional threshold of 10; the traditional rule is only a rough guide and neither excludes nor establishes over-fitting, and the below-unity calibration slope in the validation cohort indicates that some over-fitting cannot be ruled out. Fourth, the validation cohort contains only 20 events, so all validation estimates have wide confidence intervals. Fifth, the validation cohort differed systematically from the development cohort in age, interval since hysterectomy, morphology, marker positivity (16, 17) and prevalence of malignancy (Supplementary Table S2); some of this reflects changes in case mix and diagnostic practice over a 15-year period that we could not fully characterise, and exact calendar dates of surgery were not retained in the analysis dataset, precluding formal calendar-period modelling. Sixth, continuous predictors were entered linearly although the spline analysis raised the possibility of non-linearity for age. Seventh, we had no O-RADS or ADNEX scores for included patients. Finally, inter-observer agreement for the imaging predictors was not quantified. Endometriosis-associated adnexal lesions deserve particular attention, as they carry an excess risk of further gynaecological surgery, recurrence and malignant transformation (18, 19).

Future work should combine this clinical nomogram with quantitative imaging analysis, including radiomic features, and should evaluate the resulting tools prospectively in unselected patients managed both surgically and conservatively.

## 5 Conclusion

We present a seven-variable nomogram and a simplified three-variable version that estimate the probability of malignancy in adnexal masses after hysterectomy among patients already selected for surgery, with internal and temporal validation. The models are preliminary: independent external validation and formal clinical-impact assessment are required before their use can be recommended in routine practice.

## Data Availability

The original contributions presented in the study are included in the article/Supplementary Material, further inquiries can be directed to the corresponding authors.
