# Supplementary material for: Development and temporal validation of a nomogram for estimating the risk of malignancy in surgically managed adnexal masses after hysterectomy
Source: Front Med (Lausanne). 2026 Oct 5;13:1915489. doi: 10.3389/fmed.2026.1915489 (PMC13677894; doi:10.3389/fmed.2026.1915489)
Supplement: Supplementary file 1 [file Datasheet1.pdf]

SUPPLEMENTARY MATERIAL

Development and temporal validation of a nomogram for estimating the risk of malignancy in surgically managed adnexal masses after hysterectomy

Supplementary Table S1. Locked prediction equations, predictor coding and worked example

Item	Content
Panel A: full 7-variable nomogram	LP = −12.990<br>+ 0.934 × (age / 10) [age in years]<br>+ 0.699 × (BMI / 10) [BMI in kg/m²]<br>+ 0.401 × tumour size code [0 = <5 cm; 1 = 5–<10 cm; 2 = ≥10 cm]<br>+ 0.568 × cystic components [0 = cystic; 1 = cystic-solid / mural papilla; 2 = solid]<br>+ 1.292 × enhancement / blood flow [0 = none; 1 = dotted / mild; 2 = enriched / strong]<br>+ 3.970 × elevated CA125 [0 = <70 U/mL; 1 = ≥70 U/mL]<br>+ 0.373 × (cyst duration / 5) [duration in years]
Panel B: simplified 3-variable nomogram	LP = −3.959<br>+ 0.636 × cystic components<br>+ 1.002 × enhancement / blood flow<br>+ 3.582 × elevated CA125<br>(coding as in Panel A)
Probability conversion	P(malignancy) = exp(LP) / (1 + exp(LP))
Worked example (7-variable model)	Patient: age 55 years, BMI 25 kg/m², 7 cm cystic-solid mass, enriched blood flow, CA125 ≥ 70 U/mL, cyst duration 4 years.<br>LP = −12.990 + 0.934 × 5.5 + 0.699 × 2.5 + 0.401 × 7 + 0.568 × 1 + 1.292 × 2 + 3.970 × 1 + 0.373 × 0.8<br>= −12.990 + 5.137 + 1.748 + 2.807 + 0.568 + 2.584 + 3.970 + 0.298 = 4.124<br>P = exp(4.124) / (1 + exp(4.124)) = 0.984 (98.4%)
Worked example (3-variable model)	Same patient: LP = −3.959 + 0.636 × 1 + 1.002 × 2 + 3.582 × 1 = −3.959 + 0.636 + 2.004 + 3.582 = 2.263<br>P = exp(2.263) / (1 + exp(2.263)) = 0.906 (90.6%)

LP = linear predictor. Continuous predictors are entered on the scales shown in brackets; the coefficient applies to one unit of that scaled variable. The intercept of the seven-variable model (−12.990) was recalibrated on the development cohort because the originally reported intercept (−6.757) was incompatible with these scalings; the regression coefficients, and hence discrimination, are unchanged. The intercepts and coefficients are identical to those in Table 2 of the main manuscript.

Supplementary Table S2. Comparison of the development and temporal validation cohorts

Characteristic	Development cohort (n = 233)	Temporal validation cohort (n = 48)	P value
Demographic features			
Age, years; median (IQR)	53.0 (48.0–59.0)	60.0 (55.5–65.2)	< 0.001 a
Age <50 years	70 (30.0)	5 (10.4)	< 0.001 b
Age 50–<60 years	105 (45.1)	19 (39.6)	
Age ≥60 years	58 (24.9)	24 (50.0)	
BMI, kg/m²; median (IQR)	25.3 (23.0–27.8)	25.9 (22.4–28.3)	0.595 a
BMI ≥28 kg/m²	53 (22.7)	13 (27.1)	0.519 b
Tumour and imaging features			
Maximum diameter <5 cm	26 (11.2)	11 (22.9)	0.010 b
Maximum diameter 5–<10 cm	129 (55.4)	16 (33.3)	
Maximum diameter ≥10 cm	78 (33.5)	21 (43.8)	
Cystic	152 (65.2)	27 (56.3)	< 0.001 b
Cystic-solid / mural papilla	15 (6.4)	16 (33.3)	
Solid	66 (28.3)	5 (10.4)	

No blood flow / enhancement	154 (66.1)	31 (64.6)	0.521 d
Dotted / mild	19 (8.2)	2 (4.2)	
Enriched / strong	60 (25.8)	15 (31.3)	
Cyst duration, years; median (IQR)	8.0 (2.0–14.0)	15.0 (10.0–19.2)	< 0.001 a
Tumour biomarker			
CA125 ≥70 U/mL	45 (19.3)	19 (39.6)	0.002 b
Histopathological outcome			
Malignant lesion	50 (21.5)	20 (41.7)	0.003 b
Benign lesion	183 (78.5)	28 (58.3)	

Values are n (%) unless stated otherwise. The temporal validation cohort comprises 48 patients (20 malignant, 28 benign); this table uses all 48 patients, consistent with Table 1 of the main text. a Mann–Whitney U test; b chi-square test (Pearson, without continuity correction); d Monte-Carlo permutation test (20,000 replicates) because expected cell counts were below five. The two periods differ systematically in age, cyst duration, morphology, marker positivity and prevalence of malignancy; these differences are reported and discussed in the manuscript rather than treated as evidence of balance.

Supplementary Table S3. Complete data dictionary for all 14 candidate predictors

Predictor	Definition	Unit	Type	Coding used in regression	Reference	In final model
Age	Age at re-operation	years	Continuous	age / 10 (coefficient per 10-year increment)	n/a	Yes (7-variable)
BMI	Body-mass index = weight / height²	kg/m²	Continuous	BMI / 10 (coefficient per 10-unit increment)	n/a	Yes (7-variable)
Gravidity	Total number of pregnancies	count	Continuous	raw count	n/a	No (LASSO)
Parity	Total number of deliveries	count	Continuous	raw count	n/a	No (LASSO)
Family history of cancer	Self-reported family history of malignancy	—	Ordinal	0 = unknown; 1 = none; 2 = positive	0	No (LASSO)
History of endometriosis	Previous clinical or surgical diagnosis of endometriosis / adenomyosis	—	Binary	0 = no; 1 = yes	0	No (LASSO)
Fallopian tubes preserved	Tubes preserved at the prior hysterectomy	—	Binary	0 = removed; 1 = preserved	0	No (LASSO)
Tumour size	Maximum diameter of the index lesion on pre-operative imaging	cm (3 ordered categories)	Ordinal	0 = <5 cm; 1 = 5–<10 cm; 2 = ≥10 cm (single linear term)	0	Yes (7-variable)
Cystic components	Morphology of the mass	—	Ordinal	0 = purely cystic; 1 = cystic-solid or mural papillary projections; 2 = solid	0	Yes (both models)
Multilocular cyst	Multilocular architecture	—	Binary	0 = absent; 1 = present	0	No (LASSO)
Accompanying findings	Additional imaging findings	—	Ordinal	0 = none; 1 = dotted / minor; 2 = metastatic disease	0	No (LASSO)

Enhancement / blood flow	Colour-Doppler flow (primary) or CT/MRI enhancement if Doppler inadequate	—	Ordinal	0 = none; 1 = dotted / mild; 2 = enriched / strong	0	Yes (both models)
Elevated tumour marker	Pre-operative serum CA125	U/mL (dichotomised)	Binary	0 = CA125 <70 U/mL; 1 = CA125 ≥70 U/mL (2 × upper limit of normal)	0	Yes (both models)
Cyst duration	Interval from first radiological detection of the mass after hysterectomy to re-operation	years	Continuous	duration / 5 (coefficient per 5-year increment)	n/a	Yes (7-variable)

HE4, CA19-9 and CEA were not included: HE4 was measured in only a subset of patients, and CA19-9 / CEA were available only when selectively requested. Unit of analysis = patient; for multiple lesions the predictors were taken from the lesion with the most suspicious ultrasonographic morphology.

Supplementary Table S4. Complete histological diagnoses by cohort

Category	Histological diagnosis	Development cohort (n = 233)	Temporal validation cohort (n = 48)
Malignant	High-grade serous carcinoma (ovarian / tubal / peritoneal)	26 (52.0)	8 (40.0)
	Endometriosis-associated ovarian carcinoma (endometrioid / clear cell)	10 (20.0)	—
	Low-grade serous carcinoma	—	2 (10.0)
	Endometrioid carcinoma	—	2 (10.0)
	Clear-cell carcinoma	—	1 (5.0)
	Borderline ovarian tumour	6 (12.0)	3 (15.0)
	Tubal adenocarcinoma (non-serous, incl. mesonephric-like)	—	3 (15.0)
	Adenocarcinoma of the vaginal vault	—	1 (5.0)
	Other malignancy (not further subclassified in source records)	8 (16.0)	—
	Total malignant	50 (100)	20 (100)
Benign	Benign teratoma	120 (65.6)	—
	Chronic pelvic inflammatory disease / inflammatory adnexal lesion	54 (29.5)	—
	Myoma or other benign lesion	9 (4.9)	—
	Serous cystadenoma	—	7 (25.0)
	Mucinous cystadenoma	—	5 (17.9)
	Seromucinous cystadenoma	—	3 (10.7)
	Endometriotic cyst	—	4 (14.3)
	Simple cyst	—	2 (7.1)
	Fibroma	—	1 (3.6)
	Struma ovarii	—	1 (3.6)
	Paraovarian / mesosalpinx cyst	—	1 (3.6)
	Brenner tumour with leiomyoma	—	1 (3.6)
	Peritoneal inclusion cyst	—	1 (3.6)

	Sex-cord stromal tumour	—	1 (3.6)
	Benign cyst, not otherwise specified	—	1 (3.6)
	Total benign	183 (100)	28 (100)

Values are n (%) within the malignant or benign group of each cohort. Validation-cohort counts refer to the 48 patients (20 malignant, 28 benign). Abbreviations in the source dataset: HGSC, high-grade serous carcinoma; EAOC, endometriosis-associated ovarian carcinoma; BOT, borderline ovarian tumour; BT, benign teratoma; CPID, chronic pelvic inflammatory disease. Borderline tumours were analysed within the malignant category because they generally require oncological surgery; their numbers are reported separately so that readers may reclassify them.

Supplementary Table S5. Complete performance metrics by cohort and model

**Metric**	**Development 7-variable**	**Development 3-variable**	**Validation 7-variable**	**Validation 3-variable**
Number of patients	233	233	48	48
Number of malignant events	50	50	20	20
AUC (95% CI)	0.959 (0.922-0.990)	0.937 (0.885-0.979)	0.905 (0.791-0.985)	0.929 (0.846-0.986)
ΔAUC, 3-variable minus 7-variable (95% CI)		-0.023 (-0.045--0.005)		0.022 (-0.040-0.097)
DeLong test (3 vs 7 variable)		Z=-2.316, p=0.0205		Z=0.648, p=0.5173
Calibration intercept (95% CI)	0.000 (-0.573-0.573)	0.000 (-0.546-0.546)	0.285 (-0.707-1.277)	1.063 (0.152-1.973)
Calibration slope (95% CI)	1.018 (0.741-1.296)	1.000 (0.746-1.254)	0.713 (0.343-1.082)	0.949 (0.472-1.425)
Brier score	0.0473	0.0528	0.1207	0.1335
— At the pre-specified threshold 0.5 —				
Malignancies missed	8/50	11/50	6/20	7/20
Benign lesions below threshold	178/183	181/183	24/28	26/28
Sensitivity (95% CI)	0.840 (0.715-0.917)	0.780 (0.648-0.872)	0.700 (0.481-0.855)	0.650 (0.433-0.819)
Specificity (95% CI)	0.973 (0.938-0.988)	0.989 (0.961-0.997)	0.857 (0.685-0.943)	0.929 (0.774-0.980)
PPV (95% CI)	0.894 (0.774-0.954)	0.951 (0.839-0.987)	0.778 (0.548-0.910)	0.867 (0.621-0.963)
NPV (95% CI)	0.957 (0.917-0.978)	0.943 (0.900-0.968)	0.800 (0.627-0.905)	0.788 (0.622-0.893)
Accuracy (95% CI)	0.944 (0.907-0.967)	0.944 (0.907-0.967)	0.792 (0.657-0.883)	0.812 (0.681-0.898)
— At the exploratory Youden-optimal threshold —				
Threshold value	0.204	0.407	0.135	0.064
Malignancies missed	5/50	11/50	2/20	1/20
Benign lesions below threshold	174/183	181/183	23/28	23/28
Sensitivity (95% CI)	0.900 (0.786-0.957)	0.780 (0.648-0.872)	0.900 (0.699-0.972)	0.950 (0.764-0.991)
Specificity (95% CI)	0.951 (0.909-0.974)	0.989 (0.961-0.997)	0.821 (0.644-0.921)	0.821 (0.644-0.921)
PPV (95% CI)	0.833 (0.713-0.910)	0.951 (0.839-0.987)	0.783 (0.581-0.903)	0.792 (0.595-0.908)
NPV (95% CI)	0.972 (0.936-0.988)	0.943 (0.900-0.968)	0.920 (0.750-0.978)	0.958 (0.798-0.993)
Accuracy (95% CI)	0.940 (0.902-0.964)	0.944 (0.907-0.967)	0.854 (0.728-0.928)	0.875 (0.753-0.941)
— At the exploratory threshold 0.1 —				
Malignancies missed	5/50	6/50	2/20	1/20
Benign lesions below threshold	154/183	154/183	21/28	23/28
Sensitivity (95% CI)	0.900 (0.786-0.957)	0.880 (0.762-0.944)	0.900 (0.699-0.972)	0.950 (0.764-0.991)
Specificity (95% CI)	0.842 (0.782-0.887)	0.842 (0.782-0.887)	0.750 (0.566-0.873)	0.821 (0.644-0.921)
PPV (95% CI)	0.608 (0.494-0.711)	0.603 (0.488-0.707)	0.720 (0.524-0.857)	0.792 (0.595-0.908)
NPV (95% CI)	0.969 (0.929-0.986)	0.963 (0.921-0.983)	0.913 (0.732-0.976)	0.958 (0.798-0.993)
Accuracy (95% CI)	0.854 (0.803-0.894)	0.850 (0.798-0.890)	0.812 (0.681-0.898)	0.875 (0.753-0.941)

Development cohort n = 233 (50 malignant); temporal validation cohort n = 48 (20 malignant). AUC, ΔAUC and calibration confidence intervals were obtained from 2,000 bootstrap resamples; binomial proportions use Wilson intervals. ΔAUC and the DeLong test compare the 3-variable with the 7-variable model within each cohort. The Youden-optimal and 0.1 thresholds are exploratory and are not recommended decision boundaries. Calibration intercept = calibration-in-the-large (0 = perfect average calibration).

Supplementary Figures

Supplementary Figure S1. STARD 2015 flow diagram of patient selection, exclusion and allocation to the development and temporal validation cohorts.

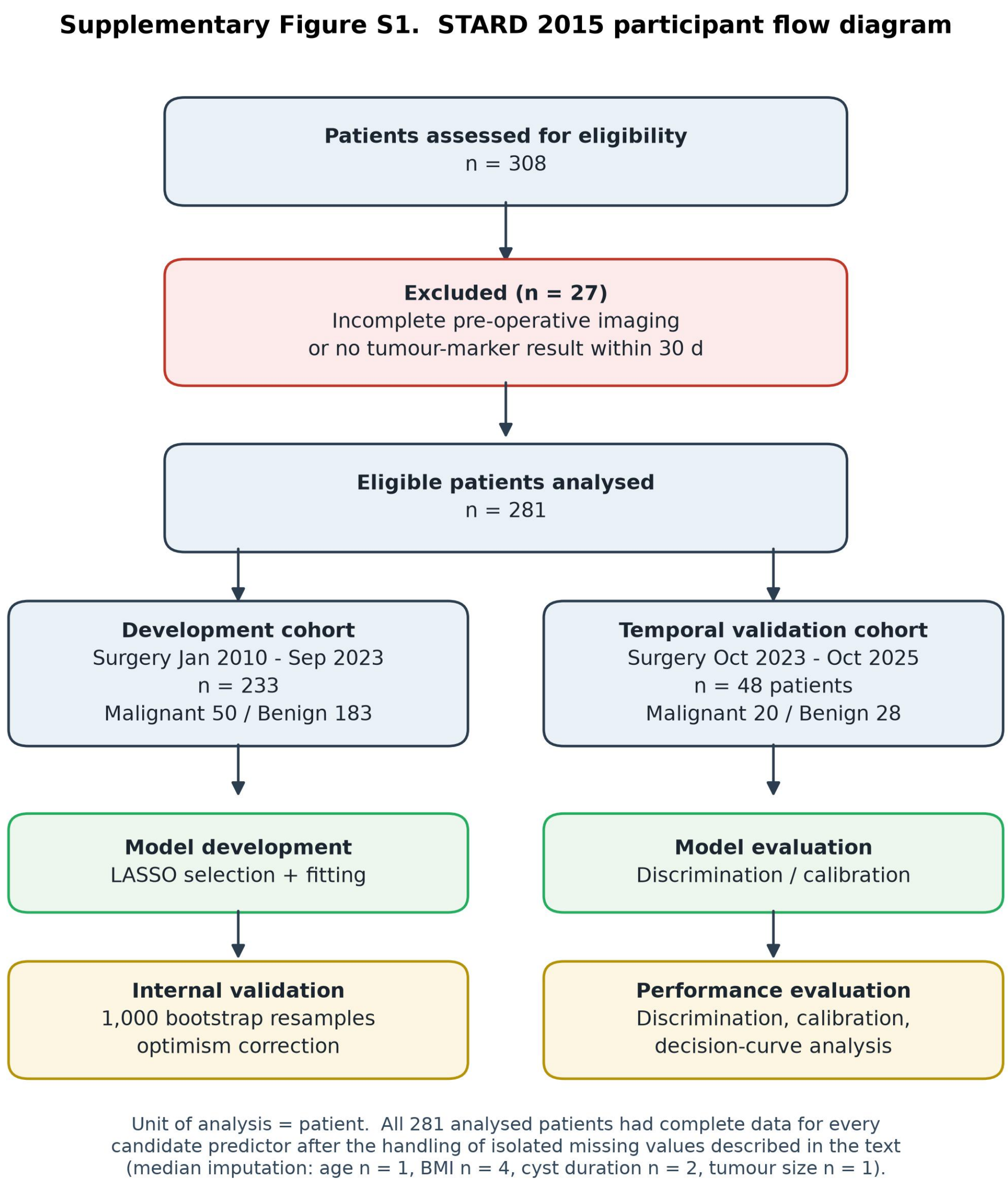

Supplementary Figure S2. Assessment of the linearity assumption (development cohort, n = 233; 50 malignant), adjusted for the remaining predictors of the seven-variable model. (A) age, (B) BMI and (C) cyst duration fitted with restricted cubic splines (four knots at the 5th, 35th, 65th and 95th centiles); solid lines are adjusted log odds ratios relative to the median, shaded areas are 95% confidence intervals, dashed lines are the pre-specified linear terms and the tick marks show the data distribution. (D) tumour size: adjusted odds ratios from an unconstrained (dummy) fit with 95% confidence intervals versus the constant odds ratio per category step implied by the ordinal fit; observed malignant proportions are printed above each category.

Supplementary Figure S2. Assessment of the linearity assumption for continuous and ordered predictors, development cohort (n = 233, 50 malignant). Curves are adjusted for the remaining predictors of the seven-variable model.

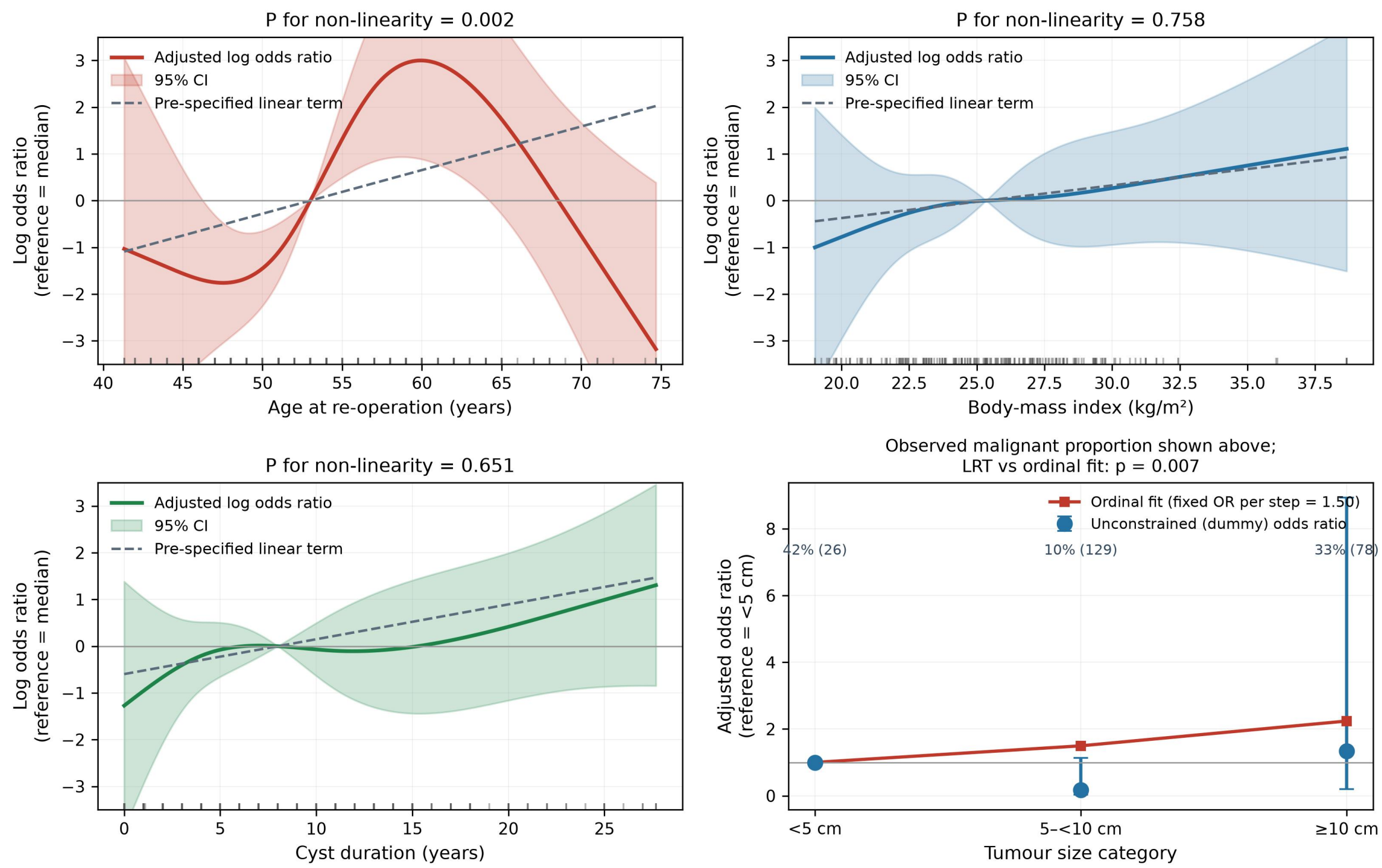

Supplementary Figure S3. Decision-curve analysis in the development cohort (n = 233; 50 malignant). Net benefit plotted against threshold probability from 0.01 to 0.89 for both models with treat-all and treat-none references. Net benefit was not standardised and confidence intervals were not computed.

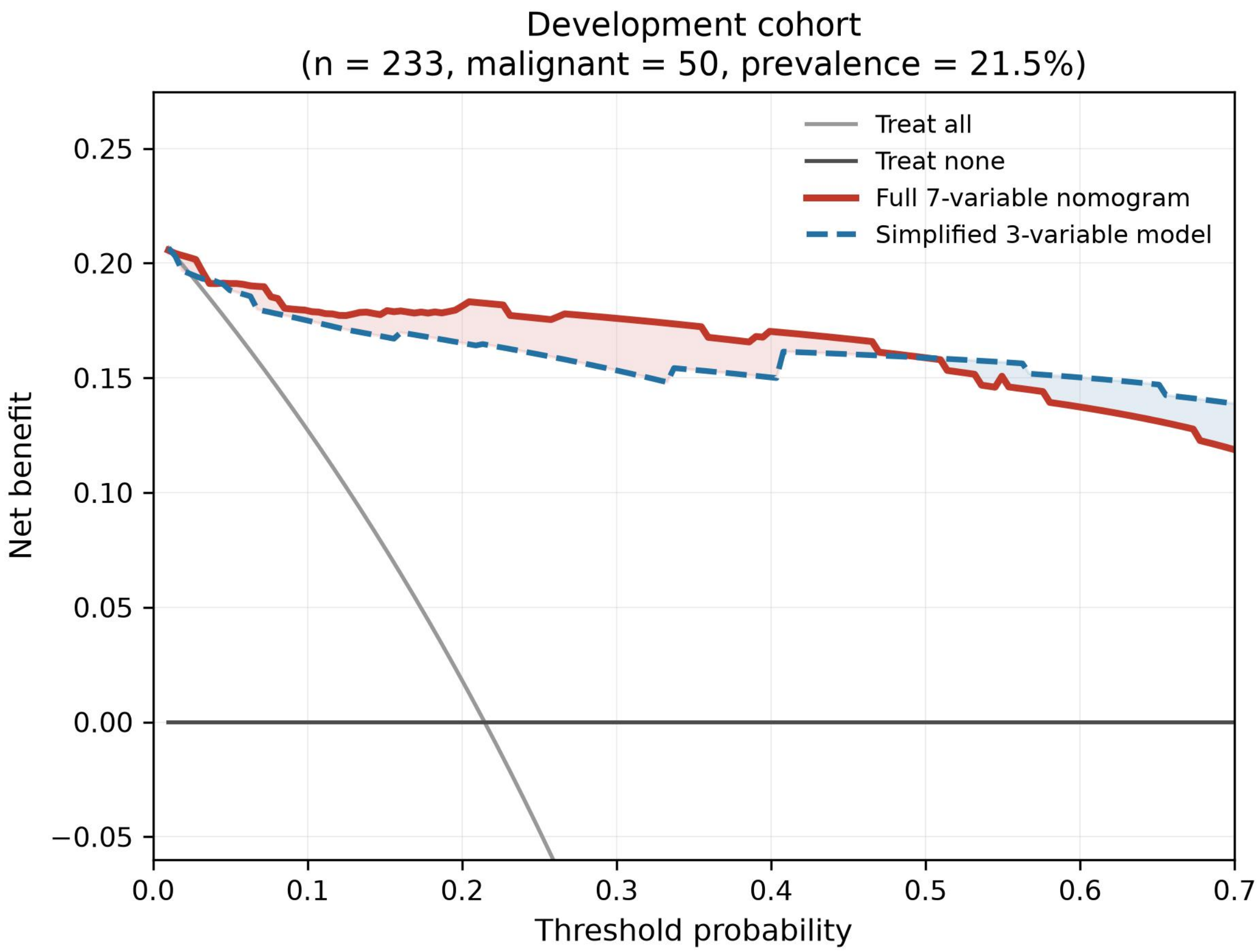

Supplementary Figure S4. Decision-curve analysis in the temporal validation cohort (n = 48; 20 malignant), presented as in Supplementary Figure S3.

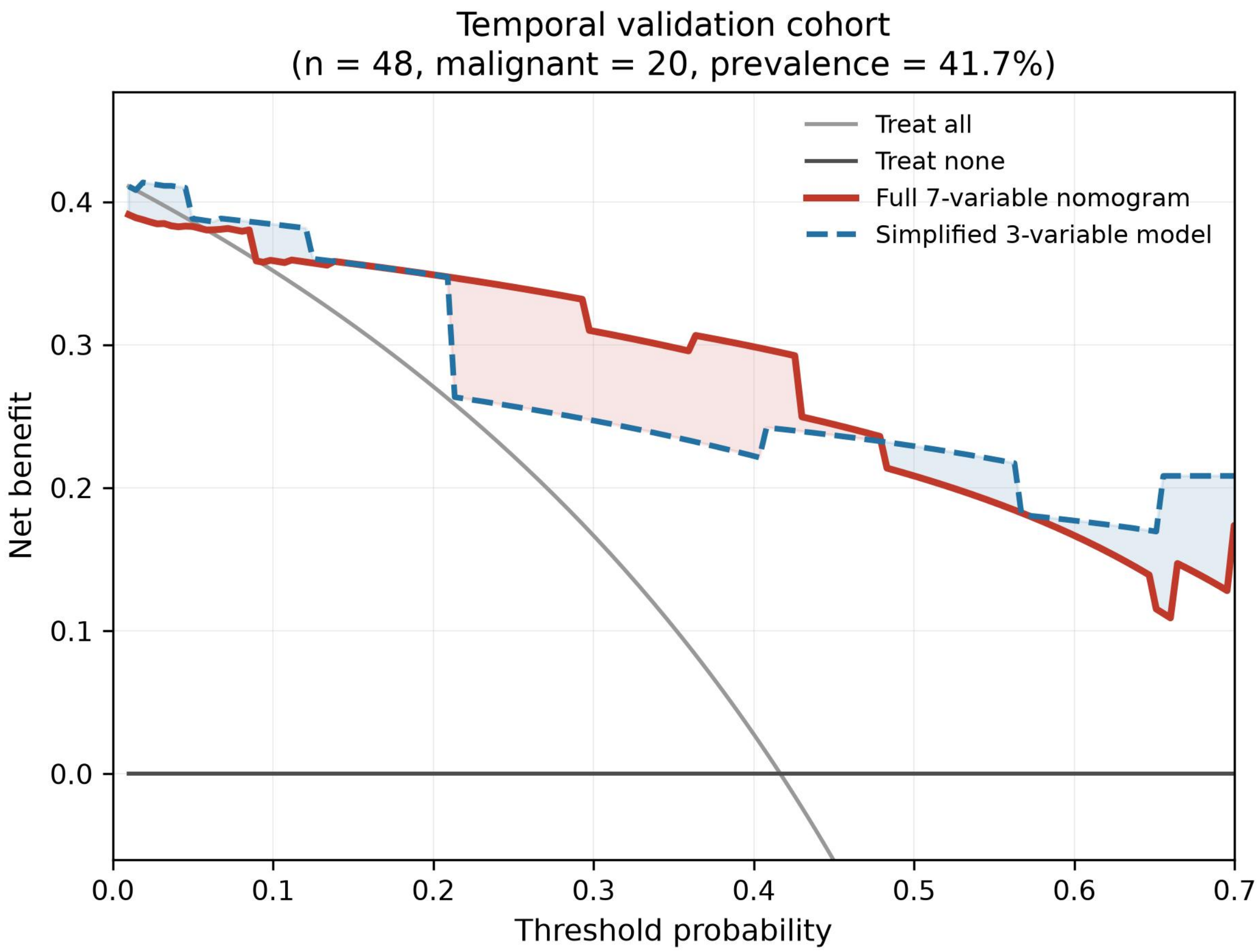

Supplementary Figure S5. Calibration of the seven-variable nomogram in the development cohort (n = 233; 50 malignant). Points are observed proportions in tenths of predicted risk with 95% Wilson confidence intervals, the thin blue line connects the deciles to guide the eye, the dashed line is perfect calibration and the rug shows the distribution of predicted probabilities. Calibration intercept 0.00 (95% CI −0.57 to 0.57); slope 1.02 (0.74 to 1.30); Brier score 0.047.

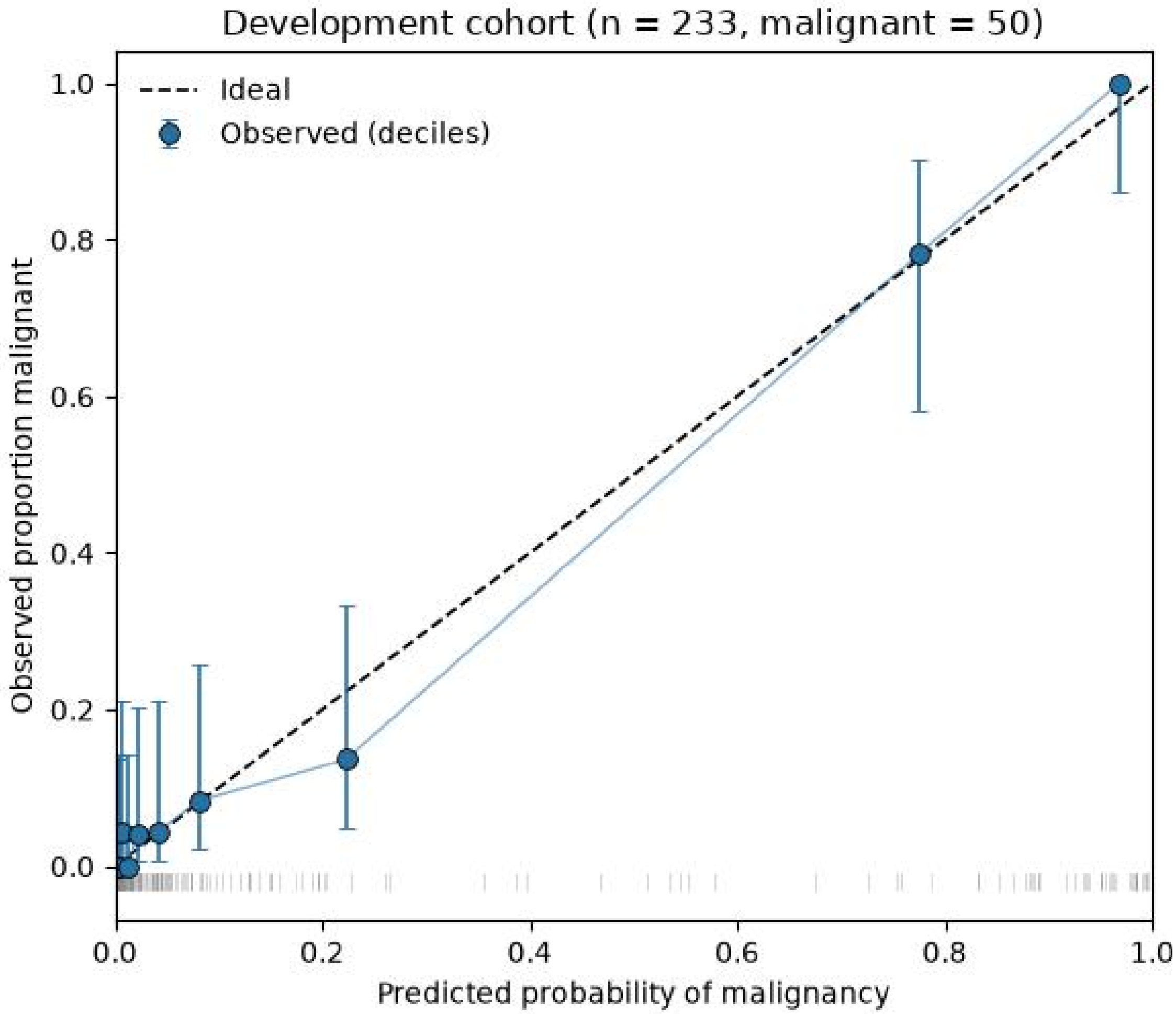

Supplementary Figure S6. Calibration of the seven-variable nomogram in the temporal validation cohort (n = 48; 20 malignant). Because of the smaller sample, observed proportions are plotted in quintiles of predicted risk with 95% Wilson confidence intervals, the thin blue line connects the quintiles to guide the eye, the dashed line is perfect calibration and the rug shows the distribution of predicted probabilities. Calibration intercept 0.29 (95% CI −0.71 to 1.28); slope 0.71 (0.34 to 1.08); Brier score 0.121.

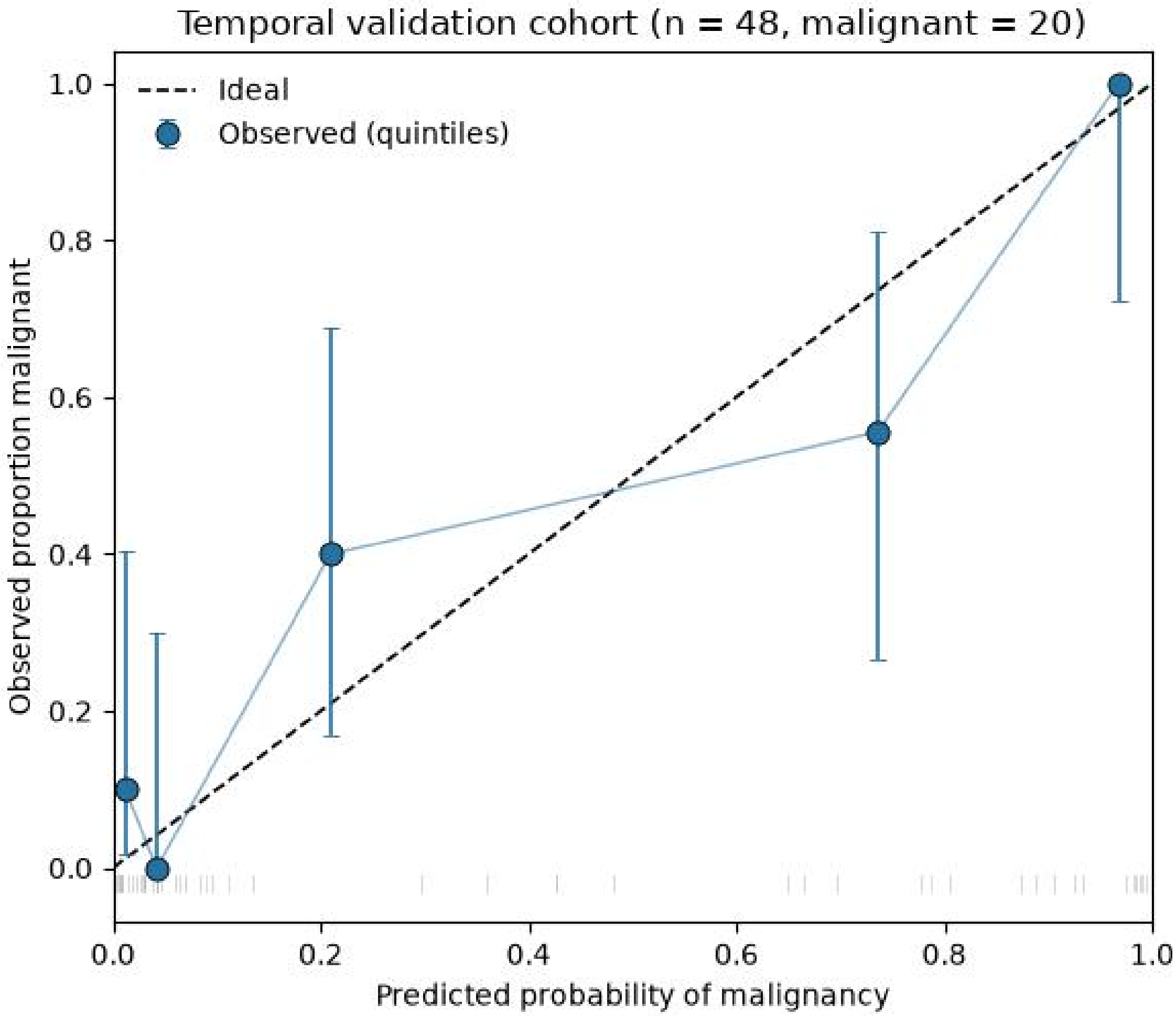

Temporal validation cohort (n = 48, malignant = 20)
Ideal
Observed (quintiles)
Observed proportion malignant
Predicted probability of malignancy
0.0
0.2
0.4
0.6
0.8
1.0
